# Anti-Adhesive Activity of Cranberry Phenolic Compounds and Their Microbial-Derived Metabolites against Uropathogenic *Escherichia coli* in Bladder Epithelial Cell Cultures

**DOI:** 10.3390/ijms160612119

**Published:** 2015-05-27

**Authors:** Dolores González de Llano, Adelaida Esteban-Fernández, Fernando Sánchez-Patán, Pedro J. Martín-Álvarez, Mª Victoria Moreno-Arribas, Begoña Bartolomé

**Affiliations:** Institute of Food Science Research (CIAL), CSIC-UAM, C/Nicolás Cabrera 9, 28049 Madrid, Spain; E-Mails: adelaida.e.fernandez@cial.uam-csic.es (A.E.-F.); f.s.patan@csic.es (F.S.-P.); pedroj.martin.alvarez@csic.es (P.J.M.-A.); victoria.moreno@csic.es (M.V.M.-A.); b.bartolome@csic.es (B.B.)

**Keywords:** UPEC, uropathogenic *Escherichia coli*, UTI, urinary tract infection, T-24 cells, phenolic metabolites, flavan-3-ols

## Abstract

Cranberry consumption has shown prophylactic effects against urinary tract infections (UTI), although the mechanisms involved are not completely understood. In this paper, cranberry phenolic compounds and their potential microbial-derived metabolites (such as simple phenols and benzoic, phenylacetic and phenylpropionic acids) were tested for their capacity to inhibit the adherence of uropathogenic *Escherichia coli* (UPEC) ATCC^®^53503™ to T24 epithelial bladder cells. Catechol, benzoic acid, vanillic acid, phenylacetic acid and 3,4-dihydroxyphenylacetic acid showed anti-adhesive activity against UPEC in a concentration-dependent manner from 100–500 µM, whereas procyanidin A2, widely reported as an inhibitor of UPEC adherence on uroepithelium, was only statistically significant (*p* < 0.05) at 500 µM (51.3% inhibition). The results proved for the first time the anti-adhesive activity of some cranberry-derived phenolic metabolites against UPEC *in vitro*, suggesting that their presence in the urine could reduce bacterial colonization and progression of UTI.

## 1. Introduction

Uropathogenic *Escherichia coli* (UPEC) is responsible for 70%–90% of urinary tract infections (UTI). On its wall, strains of uropathogenic *E. coli* have structures called fimbriae that support adhesins, by means of which they join to the receptors of the uroepithelial cells. The most common virulence factor encoding gene in the UPEC strains is the gene for type 1 fimbriae, which are important for bladder colonization with a prevalence of 89%–95%. However, the type P-fimbriae encoded by the *papG* gene are more prevalent among strains that cause invasive and persistent UTI [1,2]. The recurrence of such infections despite antibiotic treatment, as well as the morbidity associated with them, has prompted the exploration and assessment of therapeutic alternatives to the standard antibiotic treatment [3]. The prophylaxis for new and recurrent UTI by anti-adhesive compounds is coming more and more into experimental and clinical research focus. An optimized anti-adhesive entity should interact more or less specifically with adhesins of the pathogen, leading to a significant inhibition of the docking process between pro- and eukaryotic cells and further minimization of the invasion or infection of the epithelial cells [4,5].

Consumption of cranberry (*Vaccinium macrocarpum*) has been widely recommended for prophylaxis against UTI and is reputed to give symptomatic relief from these infections [6,7]. Numerous clinic studies have been carried out to evaluate the preventive effect of cranberry against UTIs, including several recent meta-analyses [8,9]. However, there is some current controversy about the clinical efficacy and cost effectiveness of cranberry supplements to decrease the risk of UTI due to the variability found in different clinical studies, although this variability has been attributed to different cranberry products and doses and lack of mechanistic guidance for the selection of subjects and study [8,10]. On the other hand, the mechanisms implied in the preventive effects of cranberry consumption against ITU are not completely established; one leading hypothesis is that cranberry components and/or their direct metabolites would operate in the phase of bacterial adherence to the uroepithelial cells, disabling or inhibiting the adherence of uropathogenic *E. coli* and, therefore, preventing bacterial colonization and progression of UTI. Moreover, cranberries are supposed to reduce UTI-related symptoms by suppressing inflammatory cascades as an immunologic response to bacteria invasion [7].

The red cranberry is rich in several groups of phenolic compounds, especially flavonols (200–400 mg/kg), anthocyanins (136–1710 mg/kg) and proanthocyanidins (PACs) (4188 mg/kg). Besides polyphenols, other phytochemicals occurring in cranberries are terpenes, organic acids, complex carbohydrates and sugars [6]. Among all of these cranberry components, A-type proanthocyanidins (PACs) seem mainly responsible for these preventive effects against UTI [6,11]. Both A- and B-type PACs are polymers of flavan-3-ol units bound through C-4 (upper unit)/C-6 or C-8 (lower unit) bonds, but only A-type PACs exhibit at least an additional ether type bond (C-2 (upper unit)-O-C-7 (lower unit)). It is known that PACs are poorly absorbed in the small intestine, reaching the colon where they are catabolized by the gut microbiota to give rise to a great battery of phenolic metabolites that can be further absorbed and also secreted in the feces [12,13]. After the intake of B-type proanthocyanidins, present in grape seeds among other sources, urine has been evidenced to contain conjugates (*i.e.*, glucuronides, *O*-methyl ethers and sulfates) of phenolic acids (*i.e.*, phenylpropionic, phenylacetic, benzoic and cinnamic acids) and other metabolites derived from the action of the microbiota, as well as other compounds derived from phase-I and phase-II metabolism, although the catabolism pathways involving these polymers are not fully elucidated [14]. Knowledge of the metabolism of A-type proanthocyanidins is less advanced [15]. Nevertheless, a recent study has reported the presence of A2-dimers in human urine after cranberry juice consumption [16]. Furthermore, some studies have reported the presence of different phenolic acids and other metabolites in urine collected after consumption of different cranberry products by healthy volunteers [16,17,18].

Cell culture methodologies have been used as an approach to studying the adherence/anti-adherence properties of *E. coli* strains in relation to UTI [3,5,19,20,21]. Studies on cell cultures have also evidenced anti-adhesive capacity against UPEC of urine samples collected after consumption of cranberry extracts [20,22,23]. Cranberry extracts and some of their components (e.g., procyanidin A2) have also been tested for *E. coli* anti-adhesive activity [3,24,25], although it is unlikely that these structures will reach the uroepithelium *in vivo*. In order to look more deeply into the bacteria anti-adhesive effects of cranberry-derived phenolic metabolites in relation to UTI, we have assessed the anti-adhesive activity against UPEC of different phenolic acids and simple phenols known to derive from the microbial catabolism of proanthocyanidins and other polyphenols and have compared these values to those corresponding to monomeric and dimeric flavan-3-ols and phenolic extracts from cranberry and grape seeds. For this, we firstly optimized an adhesion method of UPEC ATCC^®^53503™ to the T24 epithelial bladder cell line in order to improve adhesion rates in the assays. Overall, this study helps to advance the understanding of how A-type PACs are breaking down to form metabolites with *in vivo* anti-adhesion activity in the urine. This study should be considered as a first step, and further research should be done to definitively determine that the concentrations of these metabolites that have anti-adhesion activity *in vitro* are physiologically relevant *in vivo*.

## 2. Results

In order to optimize the method for evaluating the adherence of UPEC ATCC^®^53503™ to T24 epithelial cells, different initial inocula of *E. coli* (10^3^, 10^5^ and 10^8^ CFU·mL^−1^) were tested, and it was finally concluded that the value of 10^8^ CFU·mL^−1^ (1000:1 ratio of bacteria cells per epithelial cell) led to higher bacteria adherence rates (10%–14% of the total number of bacteria added initially). Using this bacteria inoculum, different incubation times of *E. coli* with phenolic extracts (from 30 min to 4 h) were tested. An incubation time of 1 h was selected in order to achieve slight improvement in the measurement of the inhibition of the adherence of UPEC ATCC^®^53503™ to T24 epithelial cells (data not shown).

**Figure 1:**
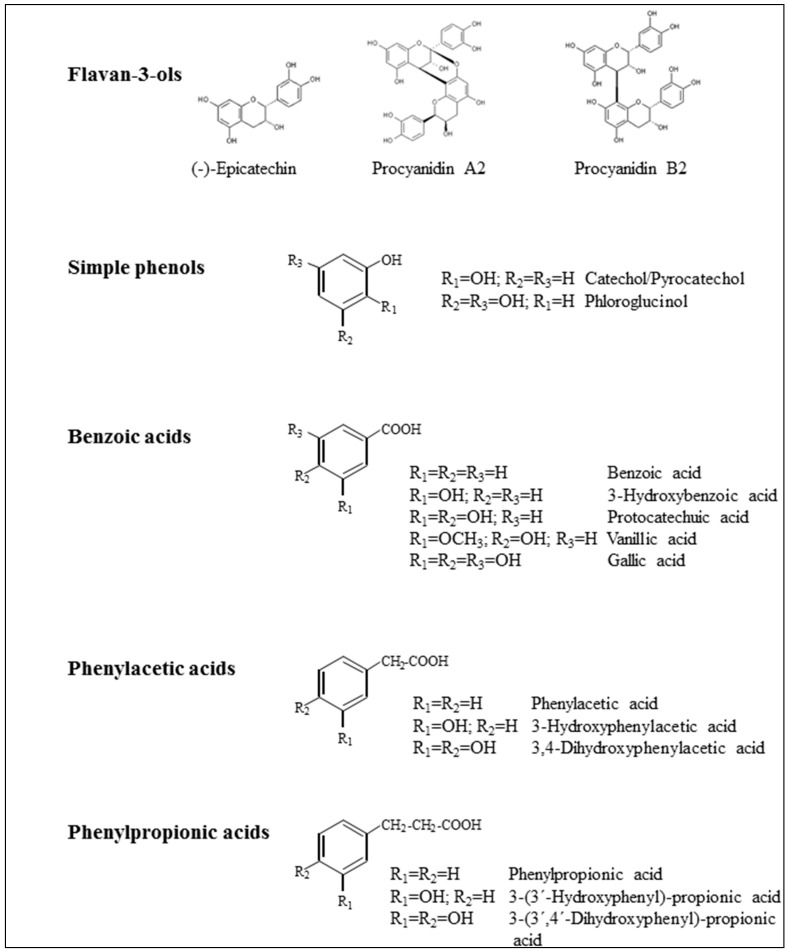
Chemical structures of phenolic compounds tested for their anti-adhesive activity against uropathogenic *Escherichia coli*.

The optimized method was applied to a total of 16 phenolic compounds, including flavan-3-ols ((−)-epicatechin) and dimeric procyanidins of A-type (A2) and B-type (B2), and microbial-derived metabolites, such as simple phenols (2), benzoic acids (5), phenylacetic acids (3) and phenylpropionic acids (3) (Figure 1). Inhibition of adherence of UPEC ATCC^®^53503™ to T24 uroepithelial cells was established by incubating constant numbers of uroepithelial cells and bacteria preincubated with phenolic compounds (at concentrations of 100, 250 and 500 µM) (Table 1). Antimicrobial and cytotoxicity activity determinations were also carried out, and the tested concentrations and times were harmless to pro- and eukaryotic cells (data not shown). In relation to flavan-3-ols, and as reported in the literature for other UPEC strains, our results showed statistically-significant (*p* < 0.05) inhibition of the adherence of UPEC ATCC^®^53503™ to bladder cells by procyanidin A2 ((−)-epicatechin-(4β-8, 2β-*O*-7)-(−)-epicatechin) (51.3% at 500 µM) (Table 1). However, no inhibitory effect was observed for procyanidin B2 ((−)-epicatechin-(4β-8)-(−)-epicatechin) or (−)-epicatechin at any of the concentrations tested (Table 1).

Once the anti-adhesive activity of A-type proanthocyanidins (*i.e.*, A2) against UPEC ATCC^®^53503™ was confirmed using our cell culture method, we tested other phenolic compounds known to derive from the microbial catabolism of proanthocyanidins and whose effect on UPEC adherence had not been studied before (Table 1). Regarding simple phenols, 1,2 dihydroxybenzene (catechol/pyrocatechol) was found to have a significant and concentration-dependent inhibitory effect against UPEC at all assayed concentrations. The same was observed for other benzoic acids (benzoic and vanillic acids) and phenylacetic acids (phenylacetic and 3,4-dihydroxyphenylacetic acids), which all exhibited inhibition percentages ≥29% at 500 µM. As seen for A2, gallic acid and 3-(3-hydroxyphenyl)-propionic acid only exhibited a significant inhibitory effect against UPEC adherence at the highest assayed concentration (500 µM).

**Table 1:** Inhibition (%) of the adherence of *E. coli* ATCC^®^53503™ to ATCC^®^HTB4™ cells by phenolic compounds.

Phenolic Compounds	C (µM)		
	100	250	500
**Flavan-3-ols**			
Procyanidin A2	7.63 ± 20.53	−5.89 ± 10.42	51.3 ** ± 7.1
Procyanidin B2	6.79 ± 22.15	10.0 ± 10.1	−14.7 ± 20.9
(−)-Epicatechin	−6.02 ± 21.95	−1.21 ± 20.76	−5.82 ± 23.28
**Simple Phenols**			
1,2-Dihydroxybenzene (catechol/pyrocatechol)	17.0 * ± 10.4	26.0 * ± 17.0	33.2 ** ± 11.7
1,3,5-Trihydroxybenzene (phloroglucinol)	−8.53 ± 10.55	17.6 ± 35.7	−8.15 ± 29.41
**Benzoic Acids**			
Benzoic acid	16.5 * ± 12.9	23.3 ** ± 14.0	32.2 ** ± 11.4
3-Hydroxybenzoic acid	11.1 ± 30.4	17.0 * ± 9.1	−9.7 ± 36.3
3,4-Dihydroxybenzoic acid (protocatechuic acid)	25.5 * ± 10.2	24.0 ± 31.8	9.44 ± 17.09
4-Hydroxy-3-methoxybenzoic acid (vanillic acid)	18.3 ** ± 2.3	24.9 ** ± 4.0	29.2 ** ± 2.2
3,4,5-Trihydroxybenzoic acid (gallic acid)	−3.72 ± 14.96	19.7 ± 43.7	40.6 ** ± 20.2
**Phenylacetic Acids**			
Phenylacetic acid	33.5 * ± 28.7	39.0 ** ± 3.3	40.6 ** ± 10.1
3-Hydroxyphenylacetic acid	15.0 ± 11.4	11.9 ± 21.8	19.4 ± 22.9
3,4-Dihydroxyphenylacetic acid	23.6 * ± 13.6	32.5 * ± 22.2	37.0 ** ± 20.5
**Phenylpropionic Acids**			
3-Phenylpropionic acid	−11.8 ± 27.2	14.7 ± 21.8	12.2 ± 13.6
3-(3-Hydroxyphenyl)-propionic acid	10.2 ± 17.6	18.6 ± 25.4	30.5 * ± 27.4
3-(3,4-Dihydroxyphenyl)-propionic acid	6.66 ± 10.63	1.19 ± 22.80	13.1 ± 15.4

* Mean significantly different from zero (*p* < 0.05) using one-sample *t*-test; ** Mean significantly different from zero (*p* < 0.01) using one-sample *t*-test.

In addition to phenolic standards, two phenolic extracts from cranberry and grape seed, respectively rich in A- and B-type proanthocyanidins, were tested for their anti-adhesive activity against UPEC ATCC^®^53503™ (Table 2). No antimicrobial and cytotoxic activity to pro- and eukaryotic cells was observed for these extracts at the tested concentrations (data not shown). In contrast to several studies that have shown an adherence inhibition for cranberry extracts [3,22,26], our results did not show significant anti-adhesive activity of either the cranberry or grape seed extracts against UPEC at concentrations of 200, 500 and 1000 mg·L^−1^ (Table 2). This was attributed to differences in the initial cranberry material and extraction procedures among extracts [27].

**Table 2:** Inhibition (%) of the adherence of *E. coli* ATCC^®^53503™ to ATCC^®^HTB4™ cells by phenolic extracts.

Phenolic Extracts	C (mg/L^−1^)		
	200	500	1000
Cranberry extract	−11.9 ± 13.0	−9.76 ± 8.65	−13.8 ± 16.5
Grape seed extract	2.21 ± 9.01	−16.4 ± 28.9	−9.8 ± 15.8

## 3. Discussion

Consumption of cranberry (*Vaccinium macrocarpum*) is widely recommended for prophylaxis against urinary tract infections (UTI) in women. Several authors have previously reported that cranberry proanthocyanidins contain unusual double A-type linkages that may be important structural features in the anti-adherence process against UPEC [20,28]. Our study agreed with these previous studies, as it showed that procyanidin A2 exhibited a statistically-significant (*p* < 0.05) inhibition of the adherence of UPEC ATCC^®^53503™ to bladder cells, but no inhibitory effect was observed for procyanidin B2. Moreover, previous comparative studies between A-type and B-type proanthocyanidins have also shown minimal or no *in vitro* anti-adhesive activity against UPEC of B-type proanthocyanidins from grape seeds and other sources, in comparison to cranberry proanthocyanidins [24]. With regard to the mechanisms involved in this anti-adhesive effect, Gupta *et al.* [25] hypothesized that A-type proanthocyanidins either combine with P-fimbriae of bacterial cells or modify the structural entity of P-fimbriae, as they observed inhibition of adherence for both sensitive and multi-drug-resistant uropathogenic P-fimbriated *E. coli* bacteria [25].

During their passage through the intestinal tract, some polyphenols are absorbed in the small intestine, although in general, they are extensively metabolized by the colon microbiota to produce phenolic metabolites, including simple phenols, phenolic acids and other metabolites [12]. Different studies have shown that phase-I and phase-II metabolites derived from the metabolism of phenolic compounds absorbed at the intestinal level are excreted in urine after the first hours of intake, whereas microbial-derived metabolites are excreted in urine at longer times [16,18]. As far as we know, our results demonstrated for the first time the *in vitro* anti-adhesive activity against UPEC of some microbial-derived phenolic metabolites, apart from A2 and other A-type proanthocyanidins, whose activity has been widely reported. Among the metabolites tested, catechol, benzoic acid, vanillic acid, phenylacetic acid and 3,4-dihydroxyphenylacetic acid showed anti-adhesive activity against UPEC in a concentration-dependent manner from 100–500 µM (Table 1). Although it was not possible to establish a consistent structure-activity relationship from the data obtained, the relevance of these findings is that phenolic acids and/or their conjugates derived from the microbial catabolism of A-type proanthocyanidins could contribute to the UPEC anti-adherence effects associated with the consumption of cranberry.

The concentrations of the phenolic metabolites tested may be physiologically relevant. From the few studies about the phenolic metabolites in human urine after cranberry consumption [16,17,18], one can work out that the urine concentration of some phenolic acids can reach at least 50 µM, which is within the range considered in our study. The free forms of phenolic acids and other phenolic compounds have been identified in plasma and urine after the intake of cranberry in humans, although conjugated phenolics are indeed dominant species [18]. Specifically, different dihydroxybenzoic acid isomers have been detected in untreated urine samples after an eight-week supplementation with cranberry capsules [17] and after a single dose of cranberry syrup [29]. As seen in this study for the free forms of certain phenolic acids, it is likely that their conjugated metabolites could inhibit UPEC adherence. As conjugated metabolites are not commercialized yet, we could not test them, but once available, future studies with glucuronidated, sulfated and methylated derivatives (for a review, see Rodriguez-Mateos *et al.* [13]) will lend valuable information about the potential of phenolic acid formulations in the prevention/treatment of UTI. Systematic protocols to synthesize phenolic conjugates are under development [30]. Another matter for further research is the mechanism(s) involved in the anti-adhesive activity against UPEC by cranberry-derived phenolics metabolites. In relation to this, Amalaradjou *et al.* [31] found that *trans*-cinnamaldehyde, a principal component in cinnamon oil, that also significantly inhibited UPEC attachment and invasion of urinary tract epithelial cells (at a concentration of 750 µM), decreased the expression of major genes involved in uropathogenic *E. coli* attachment and invasion of host tissue.

Although some particular studies showed no significant preventive effects derived from cranberry consumption [23], the general appreciation of numerous clinic studies carried out by the scientific community is that cranberry can be seen as an alternative to low-dose antibiotics in UTI prophylaxis and that additional clinical research on UTI prevention using cranberry products is clearly warranted [10].

## 4. Experimental Section

### 4.1. Phenolic Compounds, Phenolic Extracts and Other Chemicals

Flavan-3-ols ((−)-epicatechin, (−)-epicatechin-(4β-8, 2β-*O*-7)-(−)-epicatechin (procyanidin A2) and (−)-epicatechin-(4β-8)-(−)-epicatechin (procyanidin B2)) were purchased from Extrasynthèse (Genay, France). Simple phenols and phenolic acids targeted in this study (*n* = 13) are shown in Figure 1 and were obtained from Sigma-Aldrich Chemical Co. (St. Louis, MO, USA), Phytolab (Vestenbergsgreuth, Germany) or Extrasynthèse (Genay, France). Commercial powders of cranberry extract (Triarco Industries Inc. NJ, USA) and grape seed extract, Vitaflavan^®^ (Les Dérives Resiniques & Terpéniques, S.A. Dax, France), were also assayed. Total phenolic content (mg of gallic acid equivalents/g) of the cranberry and grape seed extracts was 219 and 629, respectively; as measured by the Folin-Ciocalteu reagent (Merck, Darmstadt, Germany). Phenolic composition and antioxidant activity values of these extracts were reported elsewhere [27]. The remaining chemicals and reagents were obtained from Sigma-Aldrich Co., Ltd. (Poole, Dorset, UK).

### 4.2. Preparation of Phenolic Solutions

Stock standard solutions were prepared in Dulbecco’s phosphate-buffered saline solution (DPBS, Lonza, Walkersville, MD, USA) by weighing individual phenolic compounds to achieve a starting concentration of 2500 µM, and assayed solutions were prepared via serial dilutions. Cranberry and grape seed powders were dissolved in DPBS with 0.01% and 0.1% DMSO, respectively, at a concentration of 10,000 µg·mL^−1^, and serial dilutions were prepared afterwards. All phenolic solutions were normalized to contain a maximum of 0.02% DMSO in the culture medium, which did not show any effect compared to untreated control cells.

### 4.3. Escherichia coli Strain and Growing Conditions

The UPEC ATCC^®^53503™ strain that proved to give rise to P-fimbriae [32] was used in this study. It was obtained from the American Type Culture Collection (ATCC), and bacteriological growth media (TSB and TSA broths) were obtained from Scharlau (Barcelona, Spain). *E. coli* strains that express P-fimbriae, the virulence factor most convincingly implicated in the pathogenesis of UTI, mediate Gal(α1-4)Gal-specific binding to host surfaces via the adhesin molecule PapG [2]. It was kept frozen at −70 °C in a sterilized mixture of glycerol in culture medium (20% *v*/*v*). The contents of thawed cryovials were added to the medium and grown overnight at 37 °C. Overnight cultures were harvested by centrifugation (10,000× *g*, 10 min, 4 °C) and resuspended in DPBS at a concentration of about 10^6^ and 10^8^ CFU·mL^−1^ for antibacterial and adherence assays, respectively.

### 4.4. Antibacterial Activity Assay

The antibacterial assays were performed using the method of García-Ruiz *et al.* [33]. Inhibition of *E. coli* growth by phenolic compounds and extracts was determined by the microtiter dilution method, using serial double dilutions of the antimicrobial agents and initial *E. coli* inocula (10^6^ CFU·mL^−1^). Bacteria growth was determined by reading the absorbance at 600 nm. Growth inhibitory activity was expressed as a mean percentage (%) of growth inhibition with respect to a control without antimicrobial extract. Assays were conducted in triplicate.

### 4.5. Cell Culture

We used T24 cells (ATCC^®^HTB4™), an uroepithelial cell line derived from transitional bladder carcinoma, as they have been shown to be similar to primary human bladder epithelial cells [34]. T24 bladder cells were grown and maintained in McCoy’s 5A medium (Sigma-Aldrich, St. Louis, MO, USA) supplemented with 10% (*v*/*v*) fetal bovine serum at 37 °C in an atmosphere of 5% CO_2_/95% air at constant humidity. For the experiments, cells were seeded in 96- or 24-well tissue plates and grown approximately for 24 h to enable cell attachment and to obtain a cell monolayer.

### 4.6. Cytotoxicity Assay/Cell Viability

Cytotoxicity of the tested compound against T24 cells was performed using the colorimetric 3-[4,5-dimethylthiazol-2-yl]-2,5 diphenyl tetrazolium bromide (MTT) assay [35], which is based on the reduction of the dye MTT to formazan, an insoluble intracellular blue product, by cellular dehydrogenases. Briefly, T24 cells seeded in a 96-well plate the previous day were incubated with phenolic compounds and extracts for 24 h. Then, the supernatant was removed, the monolayer washed with DPBS, and MTT was added to each well (0.5 mg·mL^−1^) and incubated for 3 h at 37 °C. Absorbance was measured at 570 nm with a plate reader (Multiskan FC Thermo Scientific, Newington, NH, USA). Assays were conducted in triplicate. The absorbance ratio between cell culture treated with the extracts and the untreated control multiplied by 100 represents cell viability (percentage of control).

### 4.7. Adherence Assay

UPEC overnight cultures (10^8^ CFU·mL^−1^) were incubated with the same volume of each phenolic compound/extract for 1 h, at 37 °C, in agitation (180 rpm). Then, confluent T24 cell monolayers (5 × 10^5^ cells/well) were washed with DPBS solution to eliminate antibiotic and overlaid with 0.5 mL of the UPEC bacteria pre-incubated with each phenolic compound/extract or with DPBS (control). After 1 h of incubation at 37 °C under 5% CO_2_ atmosphere, wells were softly washed with DPBS solution to remove unbound bacteria. Cells and adhered bacteria were then detached by trypsinization and sonicated in an ultrasonic sonication bath (3 pulses, 10 s on, 3 s off) at 40 kHz to recover bacteria associated with cells. Bacterial counts (CFU·mL^−1^) were carried out by serial dilution plate method in TSA plates. Assays were carried out in triplicate, and experiments were repeated twice. The adherence percentage (%) was calculated as the number of adhered bacteria (CFU·mL^−1^) relative to the total number of bacteria added initially × 100. The percentage of inhibition by a phenolic compound/extract was calculated as [1 − (% Adherence_sample_/% Adherence_control_)] × 100.

### 4.8. Statistical Analysis

A one-sample *t*-test was used to evaluate whether the inhibition of adherence of UPEC ATCC^®^53503™ to T24 uroepithelial cells was different from 0%. The STATISTICA program for Windows, Version 7.1 (StatSoft Inc., 1984–2006, www.statsoft.com), was used for data processing.

## 5. Conclusions

This paper reports valuable information about the inhibitory activity against UPEC adherence to uroepithelial cells by cranberry components and their potential urinary metabolites. An optimized method to study the adherence of UPEC ATCC^®^53503™ to T24 epithelial bladder cells was described. Our results demonstrated for the first time the *in vitro* anti-adhesive activity against UPEC of other low molecular weight phenolic metabolites (*i.e.*, simple phenols and phenolic acids), although it was not possible to establish a consistent structure-activity relationship from these data. This approach, considering pure standard phenolic compounds, confirms the protective action against UPEC adherence by phenolic metabolites generated from the metabolism of cranberry and, therefore, expands the beneficial effects of these metabolites in UTI prophylaxis.
